# Changes in E-cadherin associated with cytoplasmic molecules in well and poorly differentiated endometrial cancer

**DOI:** 10.1054/bjoc.2000.1386

**Published:** 2000-11

**Authors:** S Miyamoto, H Baba, S Kuroda, K Kaibuchi, T Fukuda, Y Maehara, T Saito

**Affiliations:** Gynecology Service, Gasteroenterologic Surgery, Pathology, National Kyushu Cancer Center, Notame 3-1-1, Minami-ku, Fukuoka, 811-1395; Division of Signal Transduction, Nara Institute of Science and Technology, Takayama, Ikoma, Nara, 630–0101; Department of Surgery II, Faculty of Medicine, Kyushu University, Maidashi, Higashi-ku, Fukuoka, Fukuoka, 812-8582, Japan

**Keywords:** IQGAP1, α-catenin, E-cadherin, endometrial cancer

## Abstract

E-cadherin function is thought to be impaired in epithelial cancer. To investigate the alterations in E-cadherin associated with cytoplasmic molecules including α-catenin, β-catenin, γ-catenin, p120CAS, and IQGAP1 in various endometrial cancers with different degree of differentiation, we examined the localization and expression of E-cadherin and cytoplasmic molecules in 30 cases of both well and poorly differentiated endometrioid adenocarcinomas, using immunofluorescence and immunoblotting techniques. E-cadherin and cytoplasmic molecules demonstrated linear staining at the cell boundaries in normal endometrium. In all 20 cases with well differentiated adenocarcinomas, α-catenin and IQGAP1 disappeared from the cell adhesive sites, but other cytoplasmic molecules were co-localized with E-cadherin along the cell boundaries. In all 10 cases with poorly differentiated adenocarcinomas, E-cadherin and cytoplasmic molecules accumulated as large aggregates along cell adhesive sites, and the localization of IQGAP1 differed from those of other cytoplasmic molecules. The expression of these molecules in all 20 cases with well differentiated adenocarcinomas decreased or was lost in Triton-insoluble fraction, in comparison with the findings for all cases with normal endometrium or poorly differentiated adenocarcinomas. These results suggested that each alteration in E-cadherin associated with cytoplasmic molecules may play a different role in E-cadherin dysfunction between well and poorly differentiated adenocarcinomas. © 2000 Cancer Research Campaign


*British Journal of Cancer* (2000) **83**(9), 1168–1175
© 2000 Cancer Research Campaign
doi: 10.1054/ bjoc.2000.1386, available online at http://www.idealibrary.com on IDEAL®

### Changes in E-cadherin associated with cytoplasmic molecules in well and poorly differentiated endometrial cancer

**S Miyamoto[1], H Baba[2], S Kuroda[4], K Kaibuchi[4], T Fukuda[3], Y Maehara[5] and T Saito[1]**

[1]Gynecology Service; [2]Gasteroenterologic Surgery; [3]Pathology, National Kyushu Cancer Center, Notame 3-1-1, Minami-ku, Fukuoka 811-1395; [4]Division of Signal Transduction, Nara Institute of Science and Technology, Takayama, Ikoma, Nara 630–0101; [5]Department of Surgery II, Faculty of Medicine, Kyushu University, Maidashi, Higashi-ku, Fukuoka, Fukuoka 812-8582, Japan

**Summary** E-cadherin function is thought to be impaired in epithelial cancer. To investigate the alterations in E-cadherin associated with cytoplasmic molecules including α-catenin, β-catenin, γ-catenin, p120CAS, and IQGAP1 in various endometrial cancers with different degree of differentiation, we examined the localization and expression of E-cadherin and cytoplasmic molecules in 30 cases of both well and poorly differentiated endometrioid adenocarcinomas, using immunofluorescence and immunoblotting techniques. E-cadherin and cytoplasmic molecules demonstrated linear staining at the cell boundaries in normal endometrium. In all 20 cases with well differentiated adenocarcinomas, α-catenin and IQGAP1 disappeared from the cell adhesive sites, but other cytoplasmic molecules were co-localized with E-cadherin along the cell boundaries. In all 10 cases with poorly differentiated adenocarcinomas, E-cadherin and cytoplasmic molecules accumulated as large aggregates along cell adhesive sites, and the localization of IQGAP1 differed from those of other cytoplasmic molecules. The expression of these molecules in all 20 cases with well differentiated adenocarcinomas decreased or was lost in Triton-insoluble fraction, in comparison with the findings for all cases with normal endometrium or poorly differentiated adenocarcinomas. These results suggested that each alteration in E-cadherin associated with cytoplasmic molecules may play a different role in E-cadherin dysfunction between well and poorly differentiated adenocarcinomas. © 2000 Cancer Research Campaign

**Keywords**: IQGAP1; α-catenin; E-cadherin; endometrial cancer

During embryonic development and adult life, the cadherin superfamily of cell adhesion molecules is known to be involved in many processes including cell segregation, proliferation, differentiation, and invasive behaviour (Takeichi, 1993). In human cancer, E-cadherin, which is an epithelial cell adhesion molecule, has been recognized as a powerful invasion-suppressor. It has been reported that the function of E-cadherin in zonula adherens junctions is impaired in many kinds of epithelial cancers (Shiozaki et al, 1996; Hirohashi, 1998).

Endometrial cancer, one type of epithelial cancer, is a unique epithelial cancerous state with varying levels of differentiation. In general, the oestrogen and progesterone receptors are expressed in many cases of well differentiated endometrioid adenocarcinomas, but are lacking in poorly differentiated endometrioid adenocarcinomas (Creasman, 1993). In terms of clinicopathological properties, poorly differentiated adenocarcinomas are characterized by deep myometrial invasion, high metastatic ability and a poor clinical prognosis (Kauppila et al, 1982; Creasman, 1993). In contrast, well differentiated adenocarcinomas of the endometrium have a less invasive ability, less metastatic potential, but a more favourable prognosis (Kauppila et al, 1982; Creasman, 1993). Thus, there seem to be distinct differences in cancer cells between well and poorly differentiated adenocarcinomas.

*Received 19 October 1990*
*Revised 31 May 2000*
*Accepted 31 May 2000*

*Correspondence to:* S Miyamoto

E-cadherin is a homophilic cell adhesion molecule, which depends on the presence of $Ca^{2+}$ ions. It is a transmembrane protein with five tandemly repeated extracellular domains and a cytoplasmic domain that connects the actin cytoskeleton through a complex with α-, β-, and γ-catenins. E-cadherin and cytoskeletal molecules play a crucial role in cellular morphogenesis and cell regulation, including cell shape and function (Takeichi 1993; Aberle et al, 1996; Drubin and Nelson 1996; Gumbiner, 1996). Protein p120CAS has also been defined as a member of the cadherin-based cell–cell adhesion complex, containing a series of 42 amino acid armadillo repeats, and binds directly to E-cadherin (Daniel and Reynolds, 1995). IQGAP1, as a candidate effector for the Rho family of GTPases, including Rac1 and Cdc42, can also bind β-catenin and E-cadherin (Kuroda et al, 1998). Therefore, E-cadherin function is apparently influenced by the binding of α-catenin, β-catenin, γ-catenin, p120CAS, or IQGAP1 in different cell regulating processes.

To investigate the alterations of E-cadherin associated with cytoplasmic molecules among subtypes of endometrial cancers with varying differentiation, we examined the localization and expression of E-cadherin and cytoplasmic molecules, using surgical specimens from a normal endometrium and malignant endometrial tissues including well and poorly differentiated adenocarcinomas. We found that the localization of α-catenin and IQGAP1 were disturbed in a different manner in well and poorly differentiated adenocarcinomas, compared to the findings for the normal endometrium. Comparing extracted proteins at cell adhesive sites from well differentiated adenocarcinomas, we found remarkable differences in protein expression and protein modification of E-cadherin and cytoplasmic molecules in Triton-insoluble fraction, compared to that of poorly differentiated adenocarcinomas. These results suggested that changes in E-cadherin associated with cytoplasmic molecules may be involved in the impairment of E-cadherin function in endometrial cancers with varying degrees of differentiation.

### MATERIALS AND METHODS

### Human tissues

Fresh specimens of endometrioid adenocarcinomas (20 well differentiated and 10 poorly differentiated adenocarcinomas) were obtained at surgery from 30 Japanese patients with endometrial carcinomas. The clinical features of the two types of endometrial cancers are summarized in Table 1. Normal endometrial tissue specimens were also obtained at surgery for benign disease from 4 Japanese patients in the follicular phase of the menstrual cycle. After dissection, some tissue specimens were embedded in OCT compound (Miles Laboratories, Eilhart, IN), and immediately snap-frozen in liquid nitrogen. Frozen sections were cut on a cryostat to a thickness of 4 μm, mounted on poly-L-lysine-2 coated slides, and either used immediately or stored at –80°C until needed, in order to assess the co-localization of E-cadherin and its associated cytoplasmic molecules using immunofluorescent analysis. The remaining tissue specimens dissected from the same patient were directly frozen in liquid nitrogen and also stored at –80°C, after which proteins were extracted, in order to examine the protein expression of E-cadherin and its associated cytoplasmic molecules using western blotting and tyrosine phosphorylation of β-catenin and p120CAS using immunoprecipitation.

### Immunostaining

Sections for immunostaining were fixed in PBS (phosphate-buffered saline) with 4% paraformaldehyde and 5% sucrose for one hour at room temperature, washed three times with PBS, and permeabilized for 5 min with 0.4% Triton X-100 in PBS. The sections were next washed three times with PBS, and blocked for 30 min with 5% goat serum in PBS. These sections were incubated overnight with primary antibodies at 4°C. The next day the sections were washed three times for 15 min each, incubated with secondary antibodies conjugated with fluorescein and Texas-red for 1 hour at room temperature, and then washed three times with PBS for 15 min each time. As for double staining in the use of pan-c1168adherin antibody, a solution of 150 mM NaCl, 50 mM Tris (pH 7.4), and 10 mM $CaCl_2$ was used instead of PBS. The sections were analysed by immunofluorescence microscopy, as described, using a Nikon HFX-II microscope equipped for fluorescein and Texas-red (Miyamoto et al, 1995). To acquire the assessment for distinct co-localization among E-cadherin and its associated cytoplasmic molecules, immunofluorescent signals in the cells were first magnified on a 21-inch display, overlaid, and examined using a Carl-Zweiss FISH system. For double staining, co-localization was calculated as the ratio A/B, where A equals the number of cells with an accumulation of yellow signals appearing as an immunofluorescent overlap at cell adhesive sites and B equals the number of cells with an accumulation of red signals at the cell adhesive sites. For each section, 100 cells were scored, and the scoring was repeated three times for the different fields. The sum of all three experiments was calculated. Immunostaining was repeated on at least three sections from each sample and the average percentage for the A/B ratio was recorded as the co-localization scoring. In each case, one additional section was stained with hematoxylin and eosin for verifying the presence of cancer cells and confirming the histological grading.

**Table 1** Clinical features in endometrial cancers

| | | G1 (N = 20) | G3 (N = 10) |
|---|---|---|---|
| Ages | | 54.2 ± 12.3 (30–72) | 51.3 ± 13.2 (30–66) |
| Stages | I | 14 | 5 |
| | II | 4 | 3 |
| | III | 2 | 2 |
| | IV | 0 | 0 |
| Myometrial Invasion | None | 11 | 1 |
| | ≤ 1/2 | 9 | 2 |
| | > 1/2 | 0 | 7 |
| Lymph Node Involvement | | 1 | 7 |

G1: Well differentiated adenocarcinomas, G3: Poorly differentiated adenocarcinomas.

### Immunological reagents

Antibodies against adhesion and cytoplasmic molecules, including E-cadherin, α-catenin, β-catenin, γ-catenin and p120CAS were purchased from Transduction Laboratories (Lexington, KY, USA). A polyclonal antibody against IQGAP1 was used. For the double staining of E-cadherin with other molecules, we used a pan-cadherin antibody obtained from Sigma Chemical Company (St. Louis, MO, USA). Fluorescein and Texas-red labelled secondary antibodies were obtained from BioSource International (Camarillo, CA, USA) and Molecular Probes (Eugene, OR, USA), respectively. Monoclonal antibody against phosphotyrosine was obtained from Upstate Biotechnology (Lake Placid, NY, USA). Sheep anti-mouse IgG and rabbit IgG conjugated with peroxidase (Amersham Corp, Arlington Heights, IL, USA) were used as secondary antibodies for Western immunoblotting.

### Extraction of Triton-soluble and Triton-insoluble proteins

To compare the protein expression of E-cadherin and its associated cytoplasmic molecules between Triton-soluble and Triton-insoluble components, specimens (approximately 5 × 5 × 5 mm) were solubilized in 3 ml modified CSK extraction buffer (0.5% Triton X-100, 50 mM NaCl, 300 mM sucrose, 3 mM $MgCl_2$, 0.2 U/ml aprotinin, 2 μg/ml leupeptin, 1 μg/ml pepstatin A, 2 mM PMSF (phenylmethylsulphonyl fluoride), 10 mM Pipes (1,4 piperazinediethanesulphonic acid) pH 6.2, 1 mM sodium orthovanadate, 50 mM sodium fluoride, 40 mM sodium pyrophosphate) in a sonicator for 10 sec at 4°C, using a micro-ultrasonic cell disruptor (Kontes). After centrifugation for 1 hour at 4°C, the supernatant was utilized as the Triton-soluble fraction. Pellets were re-extracted, solved completely with 3 ml RIPA containing 1% Triton X-100, 1% sodium deoxycholate, 0.1% SDS (sodium dodecyl sulphate), 150 mM NaCl, 50 mM Tris, pH 8.0, 0.2 U/ml aprotinin, 2 μg/ml leupeptin, 1 μg/ml pepstatin A, 2 mM PMSF, 1 mM sodium orthovanadate, 50 mM sodium fluoride, 40 mM sodium pyrophosphate, and then the extracted protein was used as the

© 2000 Cancer Research Campaign

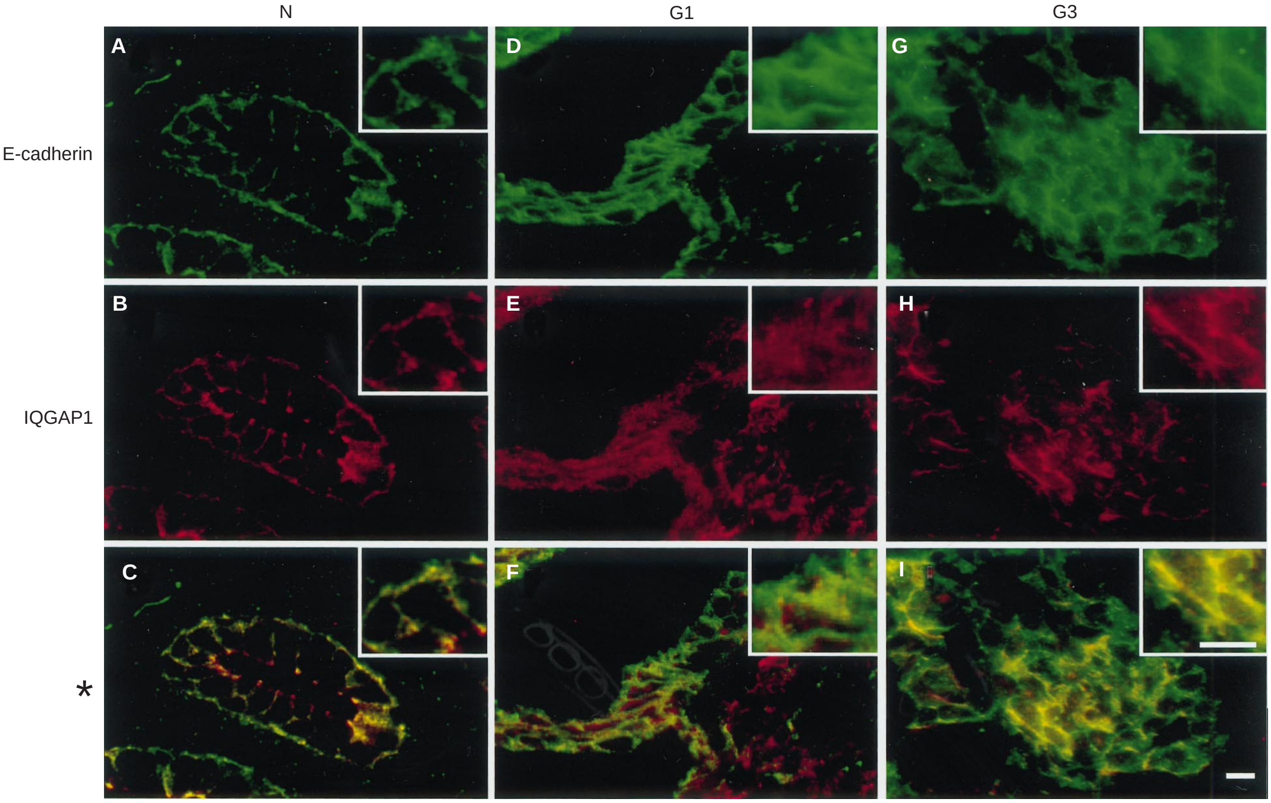

**Figure 1** The Distribution of E-cadherin and IQGAP1 in normal endometrium (N), well differentiated adenocarcinomas (G1), and poorly differentiated adenocarcinomas (G3). The localization of E-cadherin (green) or IQGAP1 (red) was determined using fluorescein or Texas-red, and immunofluorescence overlap of E-cadherin and IQGAP1 was observed as a yellow signal (*). The localization of E-cadherin and IQGAP1 proteins was observed at the zonula adherens junctions in normal endometrium (**A** and **B**) and well differentiated adenocarcinomas (**D** and **E**). In poorly differentiated adenocarcinomas, the immunostaining signals for E-cadherin and IQGAP1 revealed large aggregates along the cell boundary (**G** and **H**). Co-localization of E-cadherin and IQGAP1 is shown for each of the three groups (**C**, **F** and **I**). Each inset shows a higher-magnification view. Bar, 20 μm

Triton-insoluble components (Miyamoto et al, 1995). The protein concentrations of these two fractions were determined using a modification of the method of Bradford (Pierce Chemical, Rockford, IL, USA) (Bradford, 1976). The extracted protein ratios (mean ± standard deviation) between the Triton-insoluble components and the Triton-soluble components (Triton-insoluble components/Triton-soluble components) were 0.64 ± 0.21 (normal endometrial tissues), 0.60 ± 0.22 (well differentiated adenocarcinomas), and 0.65 ± 0.18 (poorly differentiated adenocarcinomas).

### Immunoblotting and immunoprecipitation

An equivalent amount (30 μg for the Triton-insoluble components and 70 μg for the Triton-soluble components) of each fraction was subjected to SDS-PAGE and was analysed by Western immunoblotting. To examine the tyrosine phosphorylation of β-catenin and p120CAS, immunoprecipitation was performed for the protein (1000 μg) of each fraction, by using 5 μg anti-β-catenin and 5 μg anti-p120CAS antibodies, and the precipitated samples were applied to SDS-PAGE and analysed by Western immunoblotting, using an anti-phosphotyrosine antibody, and then the same immunoblot was re-analysed with the appropriate antibodies.

### Statistical analysis

Comparisons of co-localization scorings among the three groups were made using the Bonferroni's *t*-test. $P$ values $< 0.05$ were interpreted as indicating a significant difference.

### RESULTS

### Co-localization of E-cadherin and its associated cytoplasmic molecules including α-catenin, β-catenin, γ-catenin, p120CAS, and IQGAP1

To investigate the co-localization between E-cadherin and its associated cytoplasmic molecules, double immunostaining for E-cadherin and IQGAP1, the three catenins, or p120CAS were examined in normal endometrium, well, and poorly differentiated adenocarcinomas. E-cadherin and its associated cytoplasmic molecules were expressed only in normal endometrial cells, endometrial cancer cells, and endothelial cells of vascular vessels within the tissue specimens. E-cadherin and IQGAP1 co-localized at cell adhesive sites as well as at the regions bordering stromal tissues in normal endometrium (Fig. 1A,B,C). In well differentiated adenocarcinomas, immunofluorescent signals for E-cadherin were observed along the zonula adherens junctions as a thinly broken line or a diffuse band, inside as well as outside the cancer tubules (Fig. 1D). The immunofluorescent signals for IQGAP1 revealed diffusely distributed staining in cancer cells inside and outside cancer tubules, and the localization of IQGAP1 did not coincide with that of E-cadherin (Fig. 1E, F). In poorly differentiated adenocarcinomas, E-cadherin or IQGAP1 was stained in part as large aggregates at zonula adherens junctions (Fig. 1G, H). Co-localization between E-cadherin and IQGAP1 was in part seen as abnormally clumped immunofluorescent signals along the

© 2000 Cancer Research Campaign

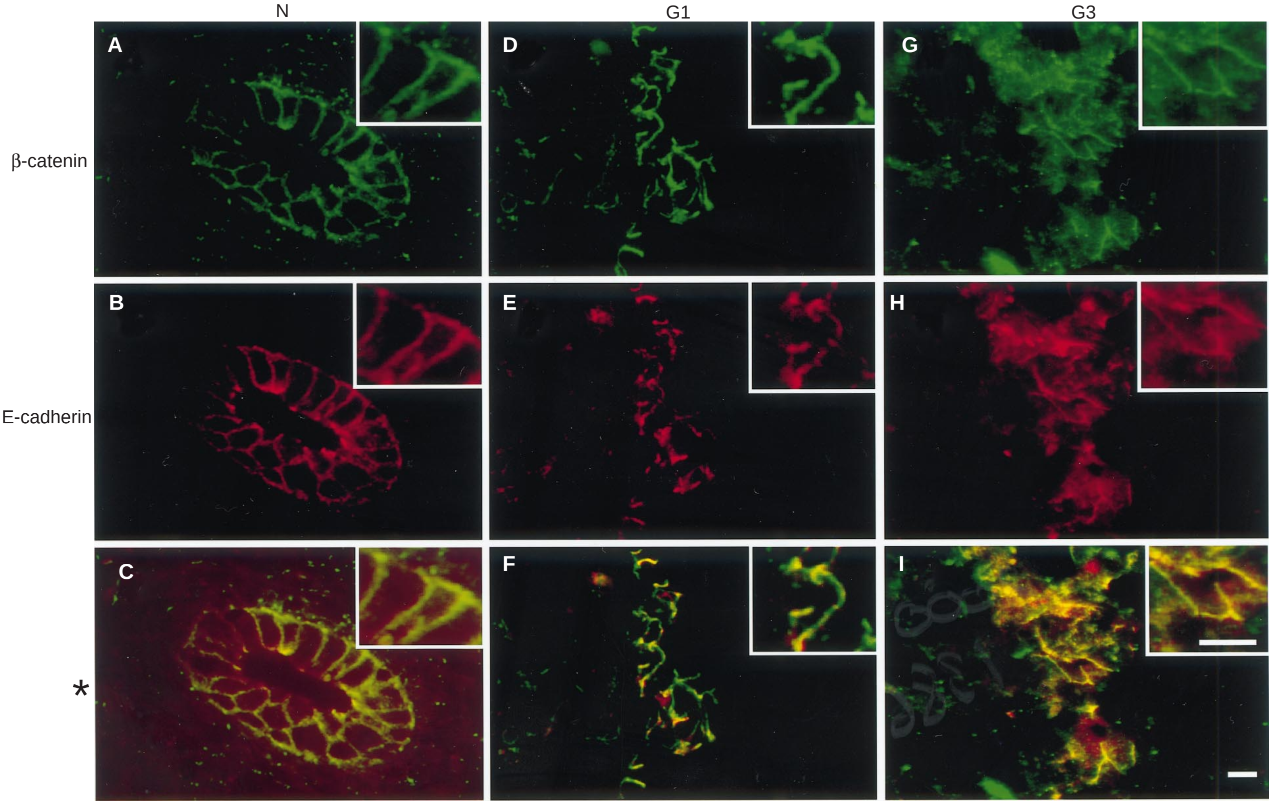

**Figure 2** The Distribution of E-cadherin and β-catenin in normal endometrium (N), well differentiated adenocarcinomas (G1), and poorly differentiated adenocarcinomas (G3). The localization of β-catenin (green) or E-cadherin (red) was determined using fluorescein or Texas-red fluorescence, and immunofluorescence overlap of β-catenin and IQGAP1 was observed as a yellow signal (*). β-catenin and E-cadherin proteins were observed at the zonula adherens junctions in normal endometrium (**A** and **B**), well differentiated adenocarcinomas (**D** and **E**), and poorly differentiated adenocarcinomas (**G** and **H**). Co-localization of β-catenin and E-cadherin is shown for each of the three groups (**C**, **F** and **I**). Each inset shows a higher-magnification view. Bar, 20 μm zonula adherens (Fig. 1I). On the other hand, E-cadherin and β-catenin accumulated linearly and completely co-localized at the zonula adherens junctions and the borders of normal endometrium (Fig. 2A,B,C). In well differentiated adenocarcinomas, immunostaining signals for β-catenin or E-cadherin were seen as a thinly broken line or a diffuse band along cell boundaries, and the localization of β-catenin coincided almost perfectly with that of E-cadherin (Fig. 2D,E,F). In poorly differentiated adenocarcinomas, accumulations of β-catenin and E-cadherin were detected, in part, at the cell boundaries, as clumped, aggregated spots (Fig. 2G,H). However, the distribution of E-cadherin was not always consistent with that of β-catenin (Fig. 2I).

To gain more insight into the distribution of each cytoplasmic molecule mediated by E-cadherin, we compared the localization of α-catenin, β-catenin, γ-catenin and p120CAS to IQGAP1 in these two types of endometrial cancers. In well differentiated adenocarcinomas, the signals for α-catenin as well as IQGAP1 were diffusely distributed, without any co-localization inside the cells (Fig. 3A,B,C). In poorly differentiated adenocarcinomas, α-catenin and IQGAP1 were in part localized along cell boundaries, but immunofluorescent signals for α-catenin did not coincide with those of IQGAP1 at all (Fig. 3D,E,F). No morphological difference between IQGAP1 positive and α-catenin positive parts was found in the sections using haematoxylin and eosin staining. The co-localization scorings of the three catenins or p120CAS with E-cadherin, and E-cadherin, the three catenins, or p120CAS with IQGAP1 are shown in Table 2. E-cadherin and IQGAP1 were usually detected in the basolateral regions of the cells in normal endometrium. The three catenins and p120CAS were sometimes localized in the basolateral regions only in normal endometrium. In all 4 cases of normal endometrium, the immunofluorescent signals for α-catenin, β-catenin, γ-catenin, p120CAS, or IQGAP1 revealed a linear accumulation at the cell boundaries, and all the E-cadherin associated with cytoplasmic molecules were positively co-localized with E-cadherin. In all 20 cases of well differentiated adenocarcinomas, the immunofluorescent signals for β-catenin, γ-catenin or p120CAS indicated thinly broken lines or diffuse bands, and α-catenin or IQGAP1 was diffusely distributed inside the cells. The co-localization scorings of β-catenin, γ-catenin, or p120CAS with E-cadherin were almost the same as those in patients with normal endometrium, whereas the co-localization scorings of α-catenin with E-cadherin and E-cadherin, the three catenins, or p120CAS with IQGAP1 significantly decreased, compared with the scorings in patients with normal endometrium and poorly differentiated adenocarcinoma because of a diffuse distribution of α-catenin and IQGAP1. In all the 10 cases of poorly differentiated adenocarcinomas, α-catenin, β-catenin, γ-catenin, p120CAS, or IQGAP1 partly accumulated along the zonula adherens junctions as clumped aggregates, and each co-localization scoring of the three catenins or p120CAS with E-cadherin significantly decreased, compared with those in normal endometrium, although only the co-localization scoring of E-cadherin with IQGAP1 was almost the same percentage as those in patients with normal endometrium. In poorly differentiated adenocarcinoma, the three catenins and p120CAS were distinctly expressed in some of the cancer cells, which had no expression of

© 2000 Cancer Research Campaign 

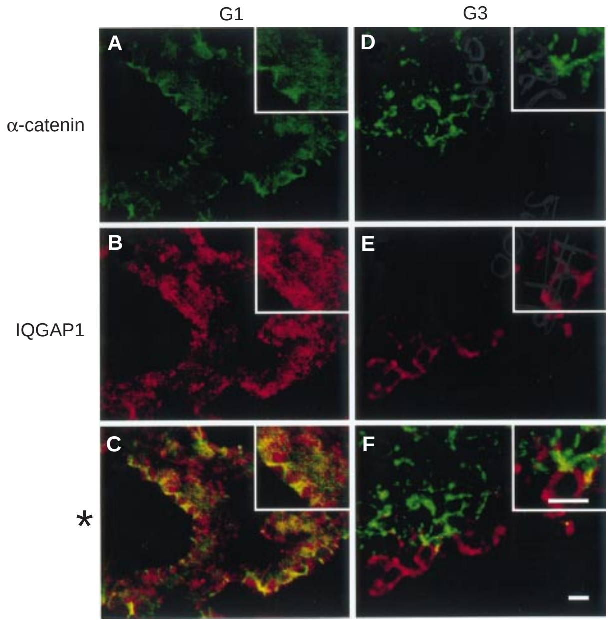

**Figure 3** The distribution of α-catenin and IQGAP1 in well differentiated adenocarcinomas (G1) and poorly differentiated adenocarcinomas (G3). The localization of α-catenin (green) or IQGAP1 (red) was determined using the fluorescein or Texas-red fluorescence, and immunofluorescence overlap of α-catenin and IQGAP1 was observed as a yellow signal (*). The diffuse expression of α-catenin (**A**) and IQGAP1 (**B**) was evident in well differentiated adenocarcinomas. As large aggregates, α-catenin and IQGAP1 proteins were observed in poorly differentiated adenocarcinomas (**D** and **E**). No immunofluorescence overlap of α-catenin and IQGAP1 was noted in the well differentiated adenocarcinomas (**C**) or in poorly differentiated adenocarcinomas (**F**). Each inset shows a higher-magnification view. Bar, 20 μm

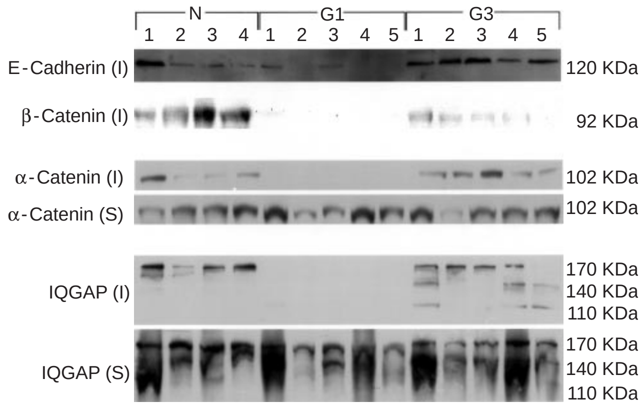

**Figure 4** The expression of E-cadherin and its associated cytoplasmic molecules in proteins of the triton-insoluble (I) and the triton-soluble fraction (S) extracted from normal endometrium (N), well differentiated adenocarcinomas (G1), and poorly differentiated adenocarcinomas (G3). In Triton-insoluble fraction (I), E-cadherin and β-catenin were expressed in normal endometrium, well differentiated adenocarcinomas, and poorly differentiated adenocarcinomas. The expression of α-catenin or IQGAP1 in the two fractions of triton-insoluble (I) and triton-soluble (S) components was detected in normal endometrium, well differentiated adenocarcinomas, and poorly differentiated adenocarcinomas. Protein extraction was independently performed at least twice for all samples. Immunoblotting was done three times for each extracted protein. The results were consistent in each experiment

IQGAP1 and little expression of the three catenins, and p120CAS was observed in the other cells in which the expression of IQGAP1 was apparently detected. However, there were no significant differences in morphological appearance between IQGAP1 positive parts and the 3 catenins or p12CAS positive parts using haematoxylin and eosin staining.

In each of the 4 cases of normal endometrium in the secretory phase or in menopause, E-cadherin and its associated cytoplasmic molecules were co-localized as a clear line at the zonula adherens junctions, just like a normal endometrium in the follicular phase. In both types of endometrial cancers, the immunofluorescent signals for β-catenin were partly detected in the cytoplasm, but no distinct accumulation of β-catenin in the nucleus was found in the normal endometrium or endometrial cancers.

### Protein expression of E-cadherin and its associated cytoplasmic molecules including α-catenin, β-catenin, γ-catenin, p120CAS, and IQGAP1

To determine if E-cadherin or its associated cytoplasmic molecules decreased or were lost at cell adhesive sites, we extracted the protein as Triton-soluble or Triton-insoluble fraction, and compared the expression of E-cadherin and cytoplasmic molecules in each fraction of all the tissues. Using the protein in Triton-insoluble fraction, the expression of E-cadherin was not detected in 3 of 5 cases with well differentiated adenocarcinomas, whereas E-cadherin was clearly expressed in all 4 cases with normal endometrium and the 5 cases with poorly differentiated adenocarcinomas (Fig. 4). The expression of β-catenin in Triton-insoluble fraction was found in all 4 cases with normal

**Table 2** Co-localization of E-cadherin and its associated cytoplasmic molecules

| | Co-localization scoring (mean ± S.D.) | | | | | |
|---|---|---|---|---|---|---|
| | E-Cadherin (P) | | | IQGAP1 (P) | | |
| | N (N = 4) | G1 (N = 20) | G3 (N = 10) | N (N = 4) | G1 (N = 20) | G3 (N = 10) |
| E-cadherin (M) | 95.2 ± 4.5 | 96.2 ± 4.5 | 95.4 ± 7.5 | 94.2 ± 3.8 | 15.9 ± 8.2 [a] | 80.2 ± 14.6 |
| α-catenin(M) | 88.2 ± 4.2 | 15.2 ± 9.5[a] | 64.5 ± 6.5[a] | 89.5 ± 5.4 | 5.2 ± 7.2[a] | 16.2 ± 8.2[a] |
| β-catenin (M) | 89.2 + 4.3 | 82.3 ± 7.0 | 62.5 ± 4.8[a] | 96.2 ± 5.2 | 17.2 ± 5.2[a] | 14.3 ± 8.9[a] |
| γ-catenin (M) | 87.5 ± 6.2 | 81.3 ± 8.2 | 59.2 ± 4.0[a] | 94.2 ± 3.2 | 18.2 ± 5.2[a] | 15.2 ± 9.1[a] |
| p120CAS (M) | 90.5 ± 4.2 | 83.2 ± 7.4 | 68.2 ± 7.0[a] | 92.2 ± 4.2 | 20.2 ± 4.2[a] | 18.2 ± 5.9[a] |

Table shows the results for double staining, using monoclonal antibodies (M) and polyclonal antibodies (P). [a]Significant decrease of co-localization scoring, compared to patients with normal endometrium. N: Normal endometrium, G1: Well differentiated adenocarcinomas, G3: Poorly differentiated adenocarcinomas.

 *© 2000 Cancer Research Campaign*

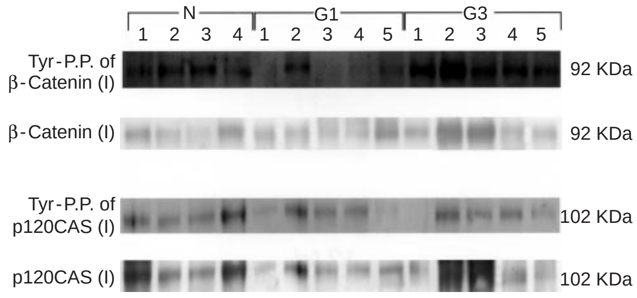

**Figure 5** Tyrosine phosphorylation of β-catenin and p120CAS in the triton-insoluble fraction (I) extracted from normal endometrium (N), well differentiated adenocarcinomas (G1), and poorly differentiated adenocarcinomas (G3). Tyrosine-phosphorylation of β-catenin (Tyr-P. P. of β-catenin) and p120CAS (Tyr-P. P of p120CAS) in the triton-insoluble fraction (I) was examined in normal endometrium, well differentiated adenocarcinomas, and poorly differentiated adenocarcinomas. Each immunoblot was re-analysed using appropriate antibodies. Proteins were extracted independently, at least twice. Immunoprecipitation and immunoblotting were done for each extracted protein, and the results were reproducible

**Table 3** Expression and protein modification of e-cadherin and its cytoplasmic molecules in Triton-Insoluble Fraction and Triton-Soluble Fraction among normal endometrium and endometrial cancers

| | The number of positive bands in Triton-insoluble fraction<br>The number of positive bands in Triton-soluble fraction | | |
|---|---|---|---|
| | **N (N = 4)** | **GI (N = 20)** | **G3 (N = 10)** |
| E-cadherin | 4/4 | 5/20 | 10/10 |
| α-catenin | 4/4 | 0/20 | 10/10 |
| β-catenin | 4/4 | 3/20 | 9/10 |
| γ-catenin | 4/4 | 4/20 | 10/10 |
| p120CAS | 4/4 | 4/20 | 10/10 |
| IQGAP1 | 4/4 | 0/20 | 10/10 |
| *β-catenin | 4/0 | 4/0 | 10/0 |
| *p120CAS | 4/4 | 17/20 | 9/10 |

*β-catenin and *p120CAS indicate tyrosine-phosphorylated β-catenin and p120CAS, respectively. N: Normal endometrium, G1: Well differentiated adenocarcinomas, G3: Poorly differentiated adenocarcinomas.

endometrium, one of 5 cases with well differentiated adenocarcinomas, and in 4 of 5 cases with poorly differentiated adenocarcinomas. To confirm the loss of other cytoplasmic molecules associated with E-cadherin, we examined the expression of α-catenin and IQGAP1 in the two fractions. In the Triton-insoluble fraction, α-catenin and IQGAP1 were clearly expressed in all 4 cases with normal endometrium and all 5 cases with poorly differentiated adenocarcinomas, but no expression of α-catenin or IQGAP1 was noted in any of the 5 cases with well differentiated adenocarcinomas. However, α-catenin and IQGAP1 in the Triton-soluble fraction were distinctly expressed in all tissue specimens (Fig. 4). Table 3 summarizes the expression of E-cadherin and its associated cytoplasmic molecules in each fraction among normal endometrium, well and poorly differentiated adenocarcinomas. In all 4 cases with normal endometrium, E-cadherin, 3 catenins, p120CAS, and IQGAP1 were distinctly expressed in both fractions. Among the 20 cases with well differentiated adenocarcinomas, the expression of E-cadherin was found in 5 cases, and in a few cases, the expression of β-catenin, γ-catenin, or p120CAS was noted in Triton-insoluble fraction. However, the expression of α-catenin or IQGAP1 in Triton-insoluble fraction was detected in none of the 20 cases. In all 10 cases of poorly differentiated adenocarcinomas, the expression of E-cadherin, α-catenin, γ-catenin, p120CAS, or IQGAP1 was distinctly found, and the expression of β-catenin was detected in 9 of 10 cases in Triton-insoluble fraction. Each expression of E-cadherin, the three catenins, p120CAS, or IQGAP1 was distinctly found in Triton-soluble fraction extracted from all the cases including the 4 with normal endometrium, 20 cases with well differentiated adenocarcinomas, and 10 cases with poorly differentiated adenocarcinomas.

### Protein modification of β-catenin and p120CAS in each fraction

To test the protein modification of cytoplasmic molecules, we examined tyrosine-phosphorylation of β-catenin and p120CAS in each fraction. In Triton-insoluble fraction, tyrosine residues of β-catenin were clearly phosphorylated in all 4 cases with normal endometrium and all 5 cases with poorly differentiated adenocarcinomas, but not in 3 of 5 cases with well differentiated adencarcinomas (Fig. 5). The tyrosine-phosphorylation of p120-CAS in Triton-insoluble fraction was detected in all 4 cases with normal endometrium, 4 of 5 cases with well differentiated adenocarcinomas, and 4 of 5 cases with poorly differentiated adenocarcinomas (Fig. 5). The results of all cases are summarized in Table 3. Using the protein in Triton-insoluble fraction, tyrosine-phosphorylation of β-catenin or p120CAS was found in all 4 cases with normal endometrium, and in 20 cases with well diffentiated adenocarcinomas, 4 cases had clear tyrosine-phosphorylation of β-catenin, and 17 cases also indicated tyrosine-phosphorylation of P120CAS. In Triton-insoluble fraction, β-catenin was strongly tyrosine-phosphorylated in all 10 cases with poorly differentiated adenocarcinomas, whereas 9 of 10 cases indicated tyrosine-phosphorylation of p120CAS. In this study, the amount of β-catenin immunoprecipitated in Figure 5 was not correlated with those in Figure 4 among the patients with well differentiated adenocarcinoma. Figure 4 shows that the amount of β-catenin included in Triton-insoluble fraction significantly decreased in patients with well differentiated adenocarcinoma, and Figure 5 shows that the tyrosine phosphorylated form of β-catenin was little detected in precipitating the same amount of β-catenin included in Triton-insoluble fraction among these patients. Thus, these results indicated that the amount of β-catenin in Triton-insoluble fraction markedly decreased, compared to those in patients with normal endometrium and that most of the β-catenin included in Triton-insoluble fraction was of a tyrosine-unphosphorylated form in patients with well differentiated adenocarcinoma. In the Triton-soluble fraction, no tyrosine-phosphorylated β-catenin was observed in any of the tissue specimens. However, tyrosine-phosphorylation of p120CAS was similarly noted in the Triton-soluble fraction of all tissue specimens.

### DISCUSSION

Cell adhesive properties mediated by E-cadherin are regulated by cytoplasmic molecules including catenins, p120CAS, and IQGAP1, as well as by small GTPases, such as Rac1, Cdc42, or Rho (Braga et al, 1997; Kuroda et al, 1997; Takaishi et al, 1997; Jou and Nelson 1998). Regarding E-cadherin and its associated cytoplasmic molecules, adhesiveness at cell adhesion sites is enhanced, when E-cadherin links to α-catenin (Nagafuchi et al,

© 2000 Cancer Research Campaign 

1991; Shimoyama et al, 1992; Ozawa and Kemler 1998). Abnormal protein modification such as tyrosine-phosphorylation of β-catenin or p120CAS induces a loss of cell adhesion mediated by E-cadherin (Matsuyoshi et al, 1992; Behrens et al, 1993; Kinch et al, 1995). On the other hand, IQGAP1 mediates the dissociation between E-cadherin and α-catenin (Kuroda et al, 1998). In human cancers, this loss of E-cadherin function has been reported to involve several different mechanisms: the down-regulation of E-cadherin expression and gene mutation, abnormality or deletion of catenins including the absence of α-catenin, and abnormal biochemical modification of cytoplasmic molecules such as tyrosine-phosphorylation of β-catenin or p120CAS (Vleminckx et al, 1991; Takeich,i 1993; Mareel et al, 1994; Kinch et al, 1995; Shiozaki et al, 1996; Hirohashi, 1998). However, little is known about changes in the localization, expression, or protein modification of E-cadherin and its associated cytoplasmic molecules in epithelial cancers with different degrees of differentiation.

Our evidence obtained in the present study shows that in the normal endometrium, the zonula adherens junctions seem to be maintained by the accumulation of E-cadherin and its associated cytoplasmic molecules including α-catenin, β-catenin, γ-catenin, p120CAS, and IQGAP1, at cell adhesive sites. In well differentiated adenocarcinomas, the complete loss of α-catenin and IQGAP1, the decrease in expression of E-cadherin, β-catenin, γ-catenin, and p120CAS, and the reduction of tyrosine-phosphorylation of β-catenin at cell adhesive sites were found, compared with findings in normal endometrium, although E-cadherin and its associated cytoplasmic molecules were expressed in Triton-soluble fraction. Taken together, these results suggest that the decrease of E-cadherin and loss of α-catenin at cell adhesivesites may induce impairment of cell adhesiveness mediated by E-cadherin in well differentiated adenocarcinomas. Since the binding of IQGAP1 to E-cadherin induces a dissociation of cell adhesive sites, the disappearance of IQGAP1 on zonula adherens junctions might compensate for the impairment of the E-cadherin function. Tyrosine-phosphorylation of β-catenin may contribute to the maintenance of E-cadherin function because β-catenin at cell boundaries was tyrosine-phosphorylated in normal endometrium. However, it remains to be elucidated as to whether or not the decrease of tyrosine-phosphorylation of β-catenin has any effect on E-cadherin function.

In some cancers including gastric and colorectal cancer, no significant correlation was observed between E-cadherin expression and distant metastasis (Kinesella et al, 1992; Oka et al, 1992; Dorudi et al, 1993; Mayer et al, 1993). Oka et al reported that the majority of primary tumours and all tumours in live metastatic sites of gastric cancer expressed the E-cadherin protein (Oka et al, 1992). Previous studies suggested the possibility that tumour cells express immunoreactive E-cadherin that does not function normally (Oka et al, 1992; Becker et al, 1994; Matsui et al, 1994). In poorly differentiated adenocarcinoma, the abnormal complexes such as E-cadherin and IQGAP1 or E-cadherin and other cytoplasmic molecules were formed at different cell adhesive sites, and the strong tyrosine phosphorylation of β-catenin in Triton-insoluble fraction was observed. These results suggest that E-cadherin might fail to function because of a complete disruption to the cytoplasmic machinery even though E-cadherin was distinctly expressed at cell adhesive sites. On the other hand, the following things are also speculated by our findings: in some cancer cells from tissues with poorly differentiated adenocarcinomas, the aggressive accumulation of IQGAP1 bound to E-cadherin may induce the disappearance of catenins and p120CAS from the cell adhesive sites and may mediate the dissociation of E-cadherin at the zonula adherens. In other cancer cells, the loss of IQGAP1 at cell adhesive sites may induce the tight binding of the catenins and p120CAS to E-cadherin, and may enhance the function of E-cadherin at the zonula adherens. In poorly differentiated adenocarcinomas, these phenomena would happen repeatedly within the cells, as well as in the tissues. In addition, the tyrosine-phosphorylation of β-catenin seemed to increase in poorly differentiated adenocarcinomas, compared with that of normal endometrium. As a result, it is possible that tyrosine-phosphorylation of β-catenin at cell adhesive sites contributes to the impairment of E-cadherin function. Thus, E-cadherin function might be impaired by completely deviating from normal cytoplasmic machinery. However, the molecular mechanism as well as the biological meaning of our findings remains unclear.

The tyrosine-phosphorylation of P120CAS had little influence on the development of endometrial cancers because there was no significant difference in the tyrosine-phosphorylation of P120CAS among patients with normal endometrium, well, and poorly differentiated adenocarcinomas. In endometrial cancer, the mutated protein of β-catenin is localized within the cells, in the nucleus as well as the cytoplasm (Kobayashi et al, 1999). In our study, immunofluorescent signals for β-catenin were partly detected in the cytoplasm as a diffuse distribution. However, a distinct accumulation of β-catenin only in the nucleus was not observed in the tissue samples. It is speculated that the mutation of β-catenin could be detected in some cases, which had a diffuse distribution of β-catenin in the cytoplasm.

The adhesive function mediated by E-cadherin is regulated by E-cadherin and its associated cytoplasmic molecules, including α-catenin, β-catenin, γ-catenin, p120CAS, IQGAP1, and by small GTPases, and is possibly associated with such tumour suppressor genes as APC and DCC or other unknown molecules (Gumbiger 1996; Braga et al, 1997; Kuroda et al, 1997; Morin et al, 1997; Ribinfeld et al, 1997; Takaishi et al, 1997; Jou and Nelson 1998). In this study, we showed that each change in cytoplasmic molecule bound to E-cadherin may be involved in endometrial cancers with varying degrees of differentiation, which are also thought to have different tumour characteristics. Therefore, an impairment of E-cadherin function in epithelial cancers may be induced by a variety of molecular mechanisms.

### ACKNOWLEDGEMENTS

We thank Dr KM Yamada (NIDCR, NIH, USA), Dr K Mekada (Institute of Life Science, Kurume University, Japan), and Mrs Linda Saza and Mr Brian Quinn for helpful discussions, and A Tahara and M Tokita for technical assistance. This work was supported in part by a grant-in-aid for cancer research from the Fukuoka Cancer Society, Fukuoka, Japan, 'Fukuoka' OBGYN Researcher's Charity Foundation Fund, a grant-in-aid for cancer research from Minister of Health and Welfare in Japan (1998 and 1999), Grant of Clinical Research Foundation in Fukuoka (1998), and the Research for Future Programs in the Japan Society for the Promotion of Science (Project No. 97L00303).

 *© 2000 Cancer Research Campaign*

### REFERENCES

Aberle H, Schwartz H and Kemler R (1996) Cadherin-catenin complex: protein interactions and their implications for cadherin function. *J Cell Biochem* **61**: 514–523

Becher KF, Atkinson MJ, Reich U, Becker I, Nekarda H, Siewert JR and Hofler H (1994) E-cadherin gene mutations provide clues to diffuse type gastric carcinomas. *Cancer Res* **54**: 3845–3852

Behrens JK, Vakaet L, Friis R, Winterhager E, Van Roy F, Mareel MM and Birchmeier W (1993) Loss of epithelial differentiation and gain of invasiveness correlates with tyrosine phosphorylation of the E-cadherin/β-catenin complex in cells transformed with a temperature-sensitive v-SRCK gene. *J Cell Biol* **120**: 757–766

Bradford MM (1976) A rapid and sensitive method for the quantitation of microgram quantities of protein utilizing the principle of protein-dye binding. *Anal Biochem* **72**: 248–454

Braga VM, Machesky LM, Hall A and Hotchin NA (1997) The small GTPases Rho and Rac are required for the establishment of cadherin-dependent cell–cell contacts. *J Cell Biol* **137**: 1421–1431

Creasman WT (1993) Prognostic significance of hormone receptors in endometrial cancer. *Cancer* **71**: 1467–1470

Daniel JM and Reynolds AB (1995) The tyrosine kinase substrate p 120cas binds directly to E-cadherin but not to the adenomatous polyposis coli protein, β, or, α-catenin. *Mol Cell Biol* **15**: 4819–4824

Dorudi S, Sheffield JP, Poulsom R, Northover JM and Hart IR (1993) E-cadherin expression in colorectal cancer. An immunocytochemical and in situ hybridization study. *Am J Pathol* **142**: 981–986

Drubin DG and Nelson WJ (1996) Origins of cell polarity. *Cell* **84**: 335–344

Gumbiner BM (1996) Cell adhesion: the molecular basis of tissue architecture and morphogenesis. *Cell* **84**: 345–357

Hirohashi S (1998) Inactivation of the E-cadherin-mediated cell adhesion system in human cancers. *Am J Pathol* **153**: 333–339

Jou TS and Nelson WJ (1998) Effects of regulated expression of mutant RhoA and Rac1 small GTPases on the development of epithelial (MDCK) cell polarity. *J Cell Biol* **142**: 85–100

Kauppila A, Kujansuu E and Vihko R (1982) Cytosol estrogen and progestin receptors in endometrial carcinoma of patients treated with surgery, radiotherapy, and progestin. Clinical correlates. *Cancer* **50**: 2157–2162

Kinch MS, Clark GJ, Der CJ and Burridge K (1995) Tyrosine phosphorylation regulates the adhesions of ras-transformed breast epithelia. *J Cell Biol* **130**: 461–71

Kinssela AR, Green B, Lepts GC, Hill CL, Bowie G and Taylor BA (1992) The role of the cell–cell adhesion molecule E-cadherin in large bowel tumour cell invasion and metastasis. *Br J Cancer* **67**: 804–809

Kobayashi K, Sagae S, Nishioka Y, Tokino T and Kudo R (1999) Mutations of the β-catenin gene in endometrial carcinomas. *Jpn J Cancer Res* **90**: 55–59

Kuroda S, Fukata M, Fujii K, Nakamura T, Izawa I and Kaibuchi K (1997) Regulation of cell–cell adhesion of MDCK cells by Cdc42 and Rac1 small GTPases. *Biochem Biophys Res Commun* **240**: 430–435

Kuroda S, Fukata M, Nakagawa M, Fujii K, Nakamura T, Ookubo T, Izawa I, Nagase T, Nomura N, Tani H, Shoji I, Matsuura Y, Yonehara S and Kaibuchi K (1998) Role of IQGAP1, a target of the small GTPases Cdc42 and Rac1, in regulation of E-cadherin-mediated cell–cell adhesion. *Science* **281**:832–835

Mareel MM, Vleminckx K, Vermeulen S, Yan G, Bracke M and Van Roy F (1994) Downregulation in vivo of the invasion-suppressor molecule E-cadherin in experimental and clinical cancer. In: *Molecular and Cellular Basis for Cell to Cell Interaction: Its Significance in Cancer*. Hirohashi S, Moses HL, Ruoslahti E, Sugimura T, Takeichi M and Terada M (eds), Proceedings of the 24th International Symposium of the Princess Takamatsu Cancer Research Fund. The Princeton Scientific Publishing Co, Inc, pp 63–80, Princeton, New Jersey, USA

Matsui S, Shiozaki H, Inoue M, Tamura S, Doki Y, Kadowaki T, Iwazawa T, Shimaya K, Nagafuchi A, Tsukita S and Mori T (1994) Immunohistochemical evaluation of alpha-catenin expression in human gastric cancer. *Virchows Arch [A] Pathol Anat Histopathol* **424**: 375–381

Matsuyoshi N, Hamaguchi M, Taniguchi S, Nagafuchi A, Tsukita S and Takeichi M (1992) Cadherin-mediated cell-cell adhesion is perturbed by v-src tyrosine phosphorylation in metastatic fibroblasts. *J Cell Biol* **118**: 703–714

Mayer B, Johnson JP, Leitl F, Jauch KW, Heiss MM, Schildberg FW, Birchmeier W and Funke I (1993) E-cadherin expression in primary and metastatic gastric cancer: down-regulation correlates with cellular dedifferentiation and glandular disintegration. *Cancer Res* **53**: 1690–1695

Miyamoto S, Teramoto H, Coso OA, Gutkind JS, Burbelo PD, Akiyama SK and Yamada KM (1995) Integrin function: molecular hierarchies of cytoskeletal and signaling molecules. *J Cell Biol* **131**: 791–805

Morin PJ, Sparks AB, Korinek V, Barker N, Clevers H, Vogelstein B and Kinzler KW (1997) Activation of β-catenin-Tcf signaling in colon cancer by mutations in β-catenin or APC. *Science* **275**: 1787–1790

Nagafuchi A, Takeichi M and Tsukita S (1991) The 102 kd cadherin-associated protein: similarity to vinculin and posttranscriptional regulation of expression. *Cell* **65**: 849–857

Oka H, Shiozaki H, Kobayashi K, Tahara H, Tamura S, Miyata M, Doki Y, Iihara K, Matsuyoshi N, Hirano S, Takeichi M and Mori T (1992) Immunohistochemical evaluation of E-cadherin adhesion molecule expression in human gastric cancer. *Virchows Arch [A] Pathol Anat Histopathol* **421**: 149–156

Ozawa M and Kemler R (1998) Altered cell adhesion activity by pervanadate due to the dissociation of α-catenin from the E-cadherin–catenin complex. *J Biol Chem* **273**: 6166–6170

Rubinfeld B, Robbins P, El-Gamil M, Albert I, Porfiri E and Polakis P (1997) Stabilization of β-catenin by genetic defects in melanoma cell lines. *Science* **275**: 1790–1792

Shimoyama Y, Nagafuchi A, Fujita S, Gotoh M, Takeichi M, Tsukita S and Hirohashi S (1992) Cadherin dysfunction in a human cancer cell line: possible involvement of loss of α-catenin expression in reduced cell-cell adhesiveness. *Cancer Res* **52**: 5770–5774

Shiozaki H, Oka H, Inoue M, Tamura S and Monden M (1996) E-cadherin mediated adhesion system in cancer cells. *Cancer* **77**: 1605–1613

Takaishi K, Sasaki T, Kotani H, Nishida H and Takai Y (1997) Regulation of cell-cell adhesion by rac and rho small G proteins in MDCK cells. *J Cell Biol* **139**: 1047–1059

Takeichi M (1993) Cadherin in cancer: implication for invasion and metastasis. *Curr Opin Cell Biol* **5**: 806–811

Vleminckx K, Vakaet LJr, Mareel M, Fiers W and van Roy F (1991) Genetic manipulation of E-cadherin expression by epithelial tumor cells reveals an invasion suppressor role. *Cell* **66**: 107–119

© 2000 Cancer Research Campaign
